# Evaluating Liver Fibrosis by Transient Elastometry in Patients With HIV-HCV Coinfection and Monoinfection

**DOI:** 10.5812/hepatmon.15426

**Published:** 2014-08-05

**Authors:** Lucia Brescini, Elena Orsetti, Rosaria Gesuita, Francesca Piraccini, Elisa Marchionni, Silvia Staffolani, Pamela Castelli, Davide Drenaggi, Francesco Barchiesi

**Affiliations:** 1 Department of Biomedical Sciences and Public Health, Clinical Infectious Diseases, Polytechnic University of Marche, Ancona, Italy; 2 Centre for Epidemiology and Biostatistics, Polytechnic University of Marche, Ancona, Italy

**Keywords:** Coinfection, Liver Fibrosis, Transient Elastometry

## Abstract

**Background:**

Due to the high efficacy of combination antiretroviral therapy (cART), the number of patients living with HIV is increasing. Chronic HCV infection has become a leading cause of non-AIDS related morbidity and mortality in patients with HIV infection.

**Objectives:**

The aim of this cross-sectional study was to identify factors associated with liver fibrosis (LF) in patients with HIV monoinfection and HIV-HCV coinfection.

**Patients and Methods:**

We analyzed LF by transient elastometry ([TE], Fibroscan) in three groups of patients (HIV, HIV-HCV and HCV) followed at the Infectious Diseases Department of University of Ancona, Italy, between October 2009 and November 2012.

**Results:**

In total, 354 adults including 98 HIV, 70 HIV-HCV and 186 HCV patients were studied. HIV-HCV patients had a longer duration of HIV (P < 0.006) and HCV (P < 0.001) infections. Additionally, they were receiving cART therapy for a longer period (P < 0.001); they had higher prevalence of lipodystrophy (P < 0.001) and higher HCV load (P = 0.004). LF was significantly more pronounced in HCV and HIV-HCV compared to HIV patients (P < 0.001). A total of 13.3%, 39.2% and 51.4% of HIV, HCV and HIV-HCV, respectively, showed a LF ≥ F2. Additionally, a severe LF (F = 4) was significantly more frequent among HIV-HCV compared to other groups. A longer exposure to didanosine, stavudine, lopinavir/ritonavir and fosamprenavir resulted in increased LF by univariate analysis (P ranging from < 0.001 to 0.007). By logistic regression analysis, the only variables significantly associated with increased LF were HCV coinfection, older age, and high AST values (P ranging from < 0.001 to 0.036).

**Conclusions:**

HCV coinfection, older age and AST were associated with LF in patients with HIV infection.

## 1. Background

Efficacy of combination antiretroviral therapy (cART) has substantially decreased morbidity and mortality rates in patients infected with human immunodeficiency virus (HIV) (1-3). Chronic HCV infection has become a leading cause of non-AIDS related morbidity and mortality in patients with HIV infection. Several studies have shown that progression of liver disease is accelerated in patients with HIV-HCV coinfection (4). Furthermore, potential hepatotoxicity of cART has been reported in both patients with HIV monoinfection and those with hepatitis coinfection (5). Particularly, NRTIs and especially didanosine (ddI) and stavudine (d4T) can directly induce mitochondrial toxicity leading to abnormal liver function (6, 7). NNRTIs such as nevirapine (NVP) and efavirenz (EFV) can cause hypersensitivity (8, 9). Other mechanisms of liver injury include cellular stress and alterations in lipid and glucose metabolism and steatosis, as with the PIs (10).

The gold standard for evaluating liver fibrosis (LF) is biopsy (11). However, it is an invasive procedure, not always well accepted by patients and not without complications (12, 13). Nowadays, LF can be easily assessed by transient elastometry (TE, Fibroscan), an innovative noninvasive procedure (14).

## 2. Objectives

This cross-sectional study was aimed to identify factors associated with LF in patients with HIV monoinfection and HIV-HCV coinfection. 

## 3. Patients and Methods

### 3.1. Patients

A total of 354 patients followed at the Infectious Diseases Outpatients Clinic of the University Hospital Umberto I°-Lancisi-Salesi of Ancona, Italy, were included in the study (October 2009 – December 2012). We included patients older than 18 years with HIV, HIV-HCV and HCV infections. LF was assessed by TE using Fibroscan (Echo-Sens, Paris, France). In this study, we used TE cut-off described by Castera et al. as F0-F1 for values between 2.5 kPa and 7.0 kPa, F > 2 for values ≥ 7.1 Kpa, F > 3 for values ≥ 9.5 kPa and F = 4 for values ≥ 12.5 kPa (the maximum cut off values for F2, F3 and F4 were respectively 9.49, 12.49 and 75 Kpa) (15). Pregnant women, patients with HBV-coinfection or other noninfectious liver diseases, patients with ascites and those received anti-HCV therapy were excluded. Additionally, patients with cART adherence < 75%, estimated using a medication adherence questionnaire adopted routinely in our clinic, were also excluded since possible antiretroviral effects on LF would not be trustworthy (16). At the time of TE measurement, clinical and analytical data of each patient were collected. Ethanol consumption of more than 50 grams daily was considered as alcohol abuse. Past and present cART were registered, including either the type of cART, total time on cART, total time on each antiretroviral drug class (i.e. NRTIs, NNRTIs, PIs and etc.) and total time on each single drug exposure. Lipodystrophy diagnosis was based on body fat changes and classified according to the Lipodystrophy Severity Grading Scale (LSGS) (17, 18). Informed consent was obtained from each patient included in the study.

### 3.2. Statistical Analysis

Continuous variables were expressed as medians and first and third quartiles and compared by using the Wilcoxon-Mann-Whitney test (comparison between the two groups) or the Kruskal-Wallis test (comparison among the three groups). Categorical variables were expressed as absolute frequencies and percentages and were compared using Chi-Square test. A multiple logistic regression analysis was performed to evaluate the effects of HCV coinfection on LF, expressed as F > 2 and considering F0-F1 as reference category. Age, gender, HIV infection length, CDC stage, current HIV load, Zenith HIV load, cART, BMI, current HCV load, aspartate aminotransferase (AST), alanine aminotransferase (ALT), gamma glutamyl transpeptidase (GGT) and lipid profile were introduced in the model as covariates. The Benjamini-Hochberg procedure was applied to correct multiple comparison effects of drugs. ODDS Ratios (Ors) were estimated by means of 95% confidence intervals. All analyses were performed using R package and the statistical significance was assessed using a probability of error of 0.05.

## 4. Results

Table 1 describes demographic, clinical and biochemical characteristics of the participants. There were 98 HIV, 70 HIV-HCV, and 186 HCV infected patients. No significant differences were found regarding age, gender and BMI distribution. Intravenous drug use (IDUs) was significantly more frequent among HIV-HCV compared to the other groups, while sexual transmission (either homosexual or heterosexual) was significantly more frequent in HIV compared to HIV-HCV or HCV. Parenteral transmission other than IDU (i.e. blood transfusion, occult parenteral transmission) was significantly more frequent in HCV. Patients with HCV (either mono- or coinfection) had higher transaminases and lower PLT counts than HIV. GGT was significantly higher in HIV-HCV compared to both monoinfected patients. Finally, the highest values of total cholesterol, LDL-cholesterol and triglycerides were found in HIV.

**Table 1.** Clinical and Demographic Characteristics of Patients With HIV, HIV-HCV, and HCV-Infections^ a,b,c^

	HIV (n = 98)	HIV−HCV (n = 70)	HCV (n = 186)	P Value
**Male, No. (%)**	67 (68.4)	46 (65.7)	116 (62.4)	0.591^ b^
**Age, y, median (Q1−Q3)**	46 (38−54)	48 (43−50)	45 (37−58)	0.845^ a^
**BMI, kg/m^2^, median (Q1−Q3)**	24.8 (22.0−26.6)	24.0 (21.0−26.0)	24.0 (22.0−27.0)	0.255^ a^
**Race, No. (%)**				
White	90 (91.8)	68 (97.1)	182 (98.4)	0.007^ b^
Asian	0 (0)	0 (0)	2 (1.1)	
Black	8 (8.2)	2 (2.9)	1 (0.5)	
**Risk factors, No. (%)**				
Homosexual	79 (80.6)	9 (12.9)	3 (1.6)	< 0.001^ b^
Heterosexual	16 (16.3)	2 (2.9)	0 (0)	
IDU	3 (3.1)	56 (80)	89 (47.8)	
Other	0 (0)	3 (4.3)	94 (50.5)	
**Ethanol consumption, > 50 g/d, No. (%)**	7 (7.1)	7 (10)	28 (15.1)	0.127^ b^
**Smokers, No. (%)**	38 (38.8)	54 (77.1)	74 (39.8)	< 0.001^ b^
**PLT, x 103/ml, median (Q1−Q3)**	212 (182.5−246)	181 (134.5−232.7)	193 (156.5−234)	0.001^ a^
**ALT, IU/mL**	25.5 (20.2−34)	56 (37.5−76)	58 (32−104)	< 0.001^ a^
		HIV vs. HIV−HCV^ c^	HIV vs. HCV^ c^	
**AST, IU/mL**	24 (19−28)	48 (32−65.5)	44 (28−73)	< 0.001^ a^
		HIV vs. HIV−HCV^ c^	HIV vs. HCV^ c^	
**GGT, IU/mL**	33.5 (19−49)	95 (35.2−141.5)	34.5 (20−81.5)	< 0.001^ a^
		HIV vs. HIV−HCV^ c^	HIV−HCV vs. HCV^ c^	
**Total cholesterol, mg/dL**	199 (181−228)	163.5 (133.8−184.8)	177 (151.5−201.5)	< 0.001^ a^
		HIV vs. HIV−HCV^ c^	HIV vs. HCV^ c^	
**HDL cholesterol, mg/dL**	46 (39−56.7)	43 (33.5−53)	51 (40−66)	0.001^ a^
			HIV−HCV vs. HCV^ c^	
**LDL cholesterol, mg/dL**	117.5 (102−147.8)	91.5 (68−117)	109 (93−133.5)	< 0.001^ a^
		HIV vs. HIV−HCV^ c^		
**Glycaemia, mg/dL**	87.5 (81−95)	88.5 (82.2−97.7)	90 (81−100)	0.541^ a^
**Triglycerides, mg/dL**	127.5 (84.2−209)	110.5 (77−171)	84 (68−114)	< 0.001^ a^
			HIV vs. HCV^c^;HIV−HCV vs. HCV^ c^	
**Lipodystrophy, No. (%)**	14 (14.3)	26 (37.1)	0 (0)	< 0.001^ b^
**Years of known HIV infection, median (Q1−Q3)**	9 (3.2−12.0)	21 (16.2−23.0)	−	< 0.001^ a^
**CDC clinical stage, No. (%)**				
A	47 (48)	17 (24.3)	−	0.006^ b^
B	28 (28.6)	33 (47.1)	−	
C	23 (23.5)	20 (28.6)	−	
**Patients with undetectable HIV load, No. (%)**	73 (74.5)	52 (74.3)	−	0.881^ b^
**Current HIV load (only for those with detectable viral load, copies/mL), median (Q1−Q3)**	573 (64−9963)	318 (80−8355.5)	−	0.990^ a^
**Nadir CD4⁺T−cell, median (Q1−Q3)**	244.5 (133−335.5)	224.5 (111.8−334.0)	−	0.395^ a^
**Zenith HIV RNA, median (Q1−Q3)**	90265 (21370−213511)	37424 (15129−100000)	−	0.022^ a^
**Current CD4⁺T−cell, median (Q1−Q3)**	593.5 (428.2−794)	547.5 (347.0−805.0)	−	0.249^ a^
**Patients receiving cART, No. (%)**	91 (92.9)	68 (97.1)	−	0.385^ b^
**Months of cART, median (Q1−Q3)**	80.5 (26−144)	145 (62.5−213.8)	−	< 0.001^ a^
**HCV genotype, No. (%)**				
1	−	33 (47.1)	91 (48.9)	0.035^ b^
2	−	1 (1.4)	23 (12.4)	
3	−	20 (28.6)	46 (24.7)	
4	−	6 (8.6)	8 (4.3)	
**Years of known HCV infection, median (Q1−Q3)**	−	16.5 (12.2−19.7)	4 (1−12)	< 0.001^ a^
**HCV load UI/mL, median (Q1−Q3)**	−	1332995 (471786−2561296)	581294 (116124−2058326)	0.004^ a^

^a^ Kruskal-Wallis test.

^b^ Chi-Square test.

^c^ P < 0.01.

### 4.1. HIV Infection Characteristics

Compared to HIV, patients with coinfection had a significantly longer history of documented HIV infection, they were receiving cART therapy for a longer period and they had higher prevalence of lipodystrophy. Zenith HIV RNA was significantly higher in HIV than HIV-HCV. CDC stage A was more frequent in HIV, while CDC stage B was more frequent in patients with coinfection. CD4 counts (either nadir or current counts) were similar in both populations. Similarly, percentages of patients receiving cART and patients with undetectable HIV load were similar in both populations.

### 4.2. HCV Infection Characteristics

Genotype 1 was the most frequent represented HCV genotype, followed by genotype 3. Genotype 2 was significantly more frequent among HCV, while genotype 4 was more frequent among HIV-HCV. Patients with coinfection had a longer duration of documented HCV infection and higher HCV load. 

### 4.3. Liver Fibrosis

Table 2 summarizes the evaluation of LF in the three groups of patients. HIV showed significantly lower TE values than the other two groups. In total, 13.3%, 39.2% and 51.4% of HIV, HCV and HIV-HCV groups showed a LF ≥ F2, respectively. Additionally, severe LF (F = 4) was significantly more frequent among HIV-HCV compared to other groups.

**Table 2.** Distribution of Transient Elastometry Values and LF Levels in HIV, HIV-HCV and HCV Infected Patients

	HIV (n = 98)	HIV-HCV (n = 70)	HCV (n = 186)	P Value
**Fibroscan, kPa, median (Q1−Q3)**	4.70 (4.00−5.95)	7.40 (5.70−13.30)	6.25 (5.10−8.85)	< 0.001 ^a^
		HIV vs. HIV−HCV ^b^	HIV vs. HCV ^b^	
**Metavir score, No. (%)**				< 0.001 ^c^
F0−F1	85 (86.7)	34 (48.6)	113 (60.8)	
F2	9 (9.2)	13 (18.6)	30 (16.1)	
F3	1 (1.0)	5 (7.1)	17 (9.1)	
F4	3 (3.1)	18 (25.7)	26 (14.0)	

^a^ Kruskal-Wallis test.

^b^ P ≤ 0.01.

^c^ Chi-Square test.

Table 3 shows the association between exposure to a single drug or cART regimens (expressed either as percentages of subjects exposed to a single drug/regimen or as length of exposure) and the presence of LF in total HIV population (patients with HIV monoinfection and HIV-HCV coinfection). Exposure to ddI, d4T, LPV/r and FPV was significantly more frequent in patients with LF F ≥ 2. Furthermore, those patients showing a LF F ≥ 2 were exposed to ddI, d4T, LPV/r and FPV for a significantly longer period than those showing LF F0-F1. Finally, prolonged exposure to cART regimens including PIs was associated with LF F ≥ 2. However, ddI and d4T were significantly more used in HIV-HCV patients than those with HIV infection (p ranging from < 0.0001 to 0.01). Therefore, only LPV/r and FPV similarly used in both populations, remained more closely associated with an increased risk of LF ≥ 2.

**Table 3.** Association Between Different cART Exposition and LF in Patients With HIV and HIV-HCV

Drugs ^a^/cART regimens	Type of drug exposure			Time of drug exposure		
	Fibrosis F0-F1 (n = 119), No. (%)	Fibrosis ≥ F2 (n = 49), No. (%)	P value	Fibrosis F0-F1 (n = 119) Months, median (Q1-Q3)	Fibrosis ≥ F2 (n = 49) Months, median (Q1-Q3)	P value ^b^
**DDI**	31 (26.1)	24 (49.0)	0.037	0 (0-1)	0 (0-39)	0.024
**ABC**	48 (40.3)	31 (63.3)	0.053	0 (0-22.5)	16 (0-42)	0.056
**FTC**	72 (60.5)	26 (53.1)	0.631	8 (0-31)	10 (0-39)	0.792
**D4T**	25 (21.0)	25 (51.0)	0.014	0 (0-0)	1 (0-44)	0.010
**3TC**	88 (73.9)	42 (85.7)	0.292	45 (0-107.5)	84 (29-121)	0.124
**TDF**	79 (66.4)	31 (63.3)	0.916	14 (0-43)	14 (0-43)	0.916
**DDC**	11 (9.2)	7 (14.3)	0.634	0 (0-0)	0 (0-0)	0.503
**AZT**	71 (59.7)	38 (77.6)	0.124	20 (0-89)	50 (2-103)	0.203
**LPV/R**	33 (27.7)	25 (51.0)	0.037	0 (0-2)	1 (0-21)	0.027
**APV**	1 (0.8)	1 (2.0)	0.916	0 (0-0)	0 (0-0)	0.634
**DAR**	20 (16.8)	6 (12.2)	0.733	0 (0-0)	0 (0-0)	0.634
**ATV**	36 (30.3)	19 (38.8)	0.544	0 (0-4)	0 (0-17)	0.363
**FPV**	7 (5.9)	11 (22.4)	0.027	0 (0-0)	0 (0-0)	0.024
**IDV**	25 (21.0)	15 (30.6)	0.429	0 (0-0)	0 (0-7)	0.420
**NFV**	14 (11.8)	13 (26.5)	0.110	0 (0-0)	0 (0-1)	0.089
**SQV**	13 (10.9)	11 (22.4)	0.203	0 (0-0)	0 (0-0)	0.124
**EFV**	43 (36.1)	18 (36.7)	0.918	0 (0-22.5)	0 (0-19)	0.916
**NVP**	41 (34.5)	13 (26.5)	0.580	0 (0-8)	0 (0-1)	0.363
**T20**	1 (0.8)	2 (4.1)	0.580	0 (0-0)	0 (0-0)	0.294
**RAL**	10 (8.4)	9 (18.4)	0.247	0 (0-0)	0 (0-0)	0.176
**MAV**	2 (1.7)	3 (6.1)	0.477	0 (0-0)	0 (0-0)	0.255
**PIs**	82 (68.9)	41 (83.7)	0.202	24 (0-57)	60 (14-117)	0.027
**NRTIs**	113 (95.0)	47 (95.9)	0.916	164 (69.5-303)	288 (152-360)	0.056
**NNRTIs**	69 (58.0)	26 (53.1)	0.792	6 (0-63)	1 (0-37)	0.503

^a^ Abbreviations: ddI, didanosine; ABC, abacavir; FTC, emtricitabine; d4T, stavudine; 3TC, lamivudine; TDF, tenofovir; ddC, zalcitabine; AZT, zidovudine; LPV, lopinavir; APV, amprenavir; DRV, darunavir; ATV, atazanavir; FPV, fosamprenavir; IDV, indinavir; NFV, nelfinavir; SQV, saquinavir; EFV, efavirenz; NVP, nevirapine; T20, enfuvirtide; RAL, raltegravir; MVC, maraviroc; PIs, protease inhibitors; NRTIs, nucleos(t)ide-inhibitors; NNRTIs, non-nucleoside inhibitors.

^b^ P values were adjusted using the Benjamini-Hochberg method.

When the effect of HCV coinfection on LF was analyzed by logistic regression analysis, adjusting for age, gender, HIV infection length, CDC stage, current HIV load, Zenith HIV load, cART, BMI, current HCV load, AST, ALT, GGT and lipid profile, the variables significantly associated with increased LF were: HCV coinfection, older age and high AST values (Table 4).

**Table 4.** Factors Associated With F > 2 Liver Fibrosis in Patients With HIV Infection

	OR	95%CI	P Value
**HCV coinfection **	4.20	1.41−13.26	0.011
**Age, y**	1.11	1.05−1.19	0.001
**AST, UI/mL**	1.03	1.01−1.05	0.004

## 5. Discussion

The aim of this study was to evaluate LF degree in three types of population: HIV, HIV-HCV, and HCV patients by TE. It has been already reported that TE is an effective technique to diagnose advanced LF/cirrhosis; also may be useful in discriminating patients with mild and no fibrosis (16). The correlation between LF assessed by TE and histology has been shown to be quite good in patients with chronic HCV infection, regardless of HIV disease (17, 19, 20). Additionally, this technique proved to be very appropriate to identify early liver damage in patients with HIV monoinfection (7).

In our study, variables significantly associated with LF in patients with HIV were HCV coinfection, older age and AST. It has been demonstrated that HIV coinfection determines the progression of HCV LF (4). Similar to literature data, we found the highest percentage of F4 LF in our group of patients with coinfection (25.7% vs. 14% [HCV], and vs. 3.1% [HIV]) (4, 21). Besides, we found that most of patients with coinfection had LF ≤ F2 (67.6%), as most of the HCV subjects (76.9%). It has been reported that patients with HIV-HCV coinfection receiving effective cART are characterized by slower LF progression (22, 23). In total, 97% of our patients with coinfection were receiving cART for a long time (a median of 145 months) and 74% had undetectable viral load and a good immunological control (median CD4/mmc of 547). Notably, 13.3% of our HIV population showed a LF ≥ F2. Literature data reported that LF ≥ F2 investigated by TE found between 1% to 14% of patients with HIV infection receiving cART (7, 24).

Older age has been repeatedly associated with advanced fibrosis either in HIV and HCV monoinfected or HIV-HCV coinfected patients (19, 21, 25). This finding confirmed in our study can be related to a longer history of infection.

Longitudinal studies conducted in patients with HIV-HCV coinfection and HCV monoinfection showed that higher transaminases values are correlated to either advanced LF or a more rapid fibrosis progression (26, 27). Our study, although not longitudinal in nature, confirmed an association between high levels of transaminases and LF in HCV population (patients with either mono or coinfection); furthermore, AST value was significantly associated with LF in patients with HIV monoinfection using logistic regression analysis.

Liver toxicity has been mainly reported in HCV and/or HBV coinfected patients treated with cART (28-32). Although we did not find any association between specific drug/time of exposure and advanced LF by logistic regression analysis, we observed that previous and long-term exposure to ddI, d4T, FPV and LPV/r were associated with LF F ≥ 2 in univariate analysis. Our data is in agreement with what previously reported by some others. In general, the effect of ddI on LF was evidenced either in patients with HIV monoinfection or HIV-HCV coinfection. A recent study by Suárez-Zarracina et al. showed that the use of ddI in patients with HIV monoinfection and HIV-HCV coinfection was independently associated with increased LF (25). Merchante et al. studied a population with HIV monoinfection and found that long-term exposure to ddI was a major cause of liver damage (7). It has been postulated that dideoxynucleotides induce steatohepatitis via mtDNA depletion (10, 24, 33, 34). Additionally, ddI might cause liver injury by endothelial damage in the portal tract predisposing to a noncirrhotic portal hypertension and nodular regenerative hyperplasia (35). Furthermore, Maida et al. reported that removal of ddI from cART regimens of patients with HIV showing unexplained liver disease, improved clinical and biochemical parameters of liver function (36). It is difficult to explain the reason why in our cases the effect of ddI was lost by logistic regression analysis. PIs are characterized by a low hepatotoxicity and generally represent the preferred drugs to treat HIV patients with liver disease (10). Therefore, the association of LPV/FPVand LF in our series of patients might represent a bias, because PIs are generally administered to coinfected patients with advanced LF.

In conclusion, HCV coinfection, older age and AST were associated with increased LF in patients with HIV infection. Of note, we demonstrated that HIV monoinfected patient with advanced LF is not a rare event. This fact suggests the use of TE, along with biochemical markers, for liver damage monitoring in not only chronic HCV infection but also HIV monoinfection. Although liver stiffness has good correlation with LF on liver biopsy, the major drawback of this study was lack of liver biopsy on studied population. Further studies are warranted to elucidate this finding.
